# Supplementary figures and images for: NFIB promotes the migration and progression of kidney renal clear cell carcinoma by regulating PINK1 transcription
Source: PeerJ. 2021 Apr 23;9:e10848. doi: 10.7717/peerj.10848 (PMC8074839; doi:10.7717/peerj.10848)

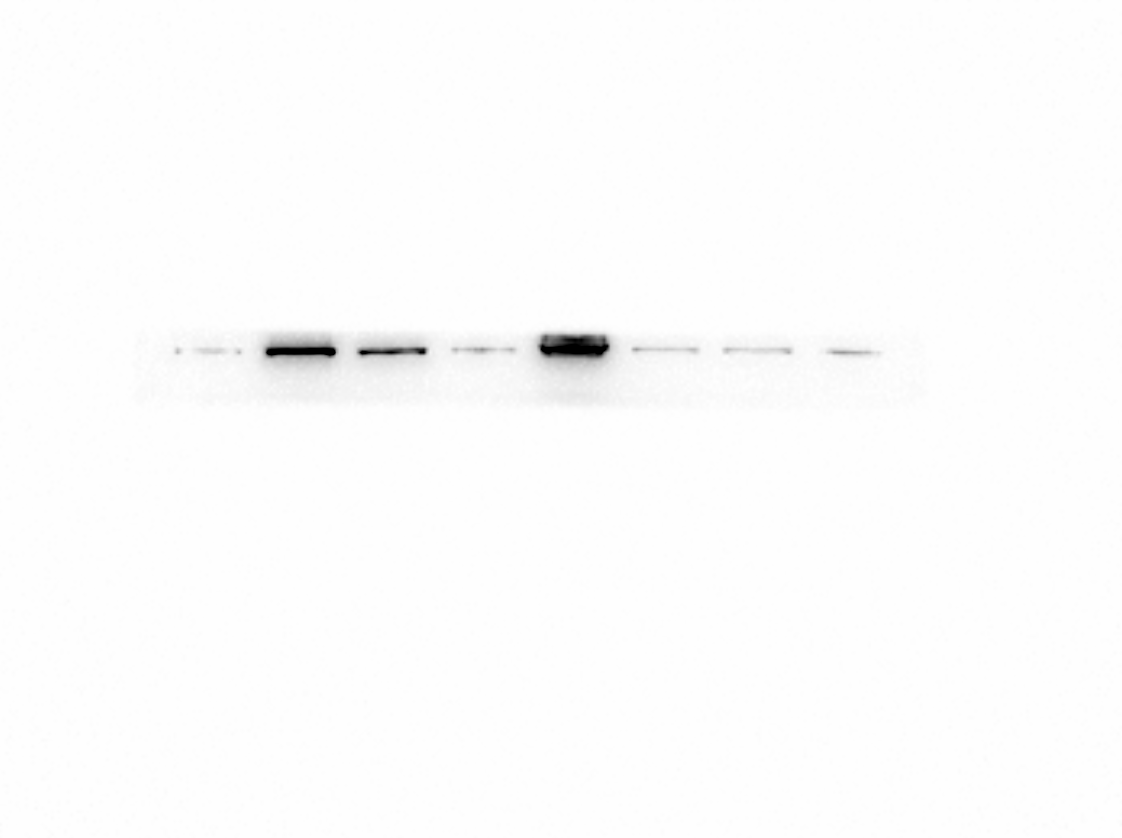

Supplement: Supplemental Information 7 [file peerj-09-10848-s007.zip › Full-length_uncropped_blots/Figure 1/NFIB.tif]

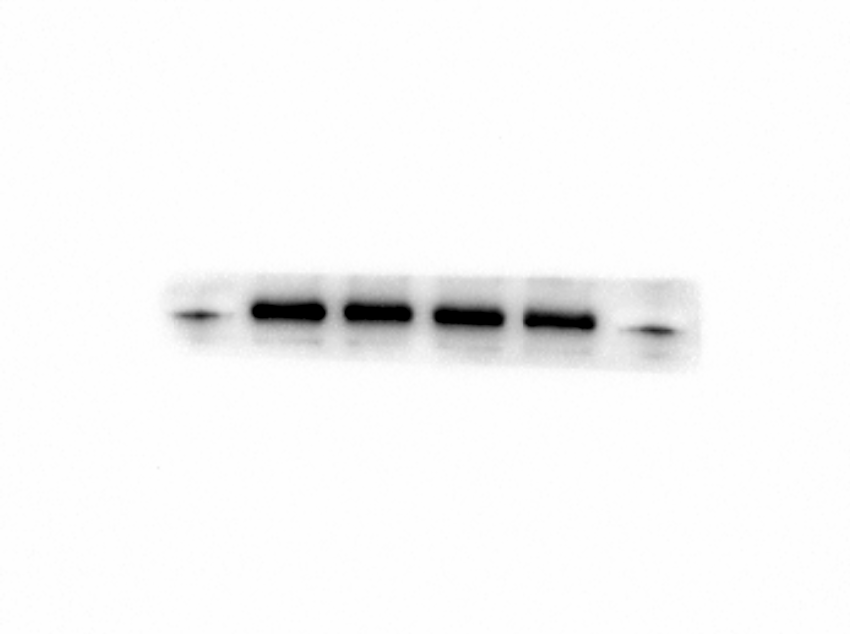

Supplement: Supplemental Information 7 [file peerj-09-10848-s007.zip › Full-length_uncropped_blots/Figure 1/GAPDH.tif]

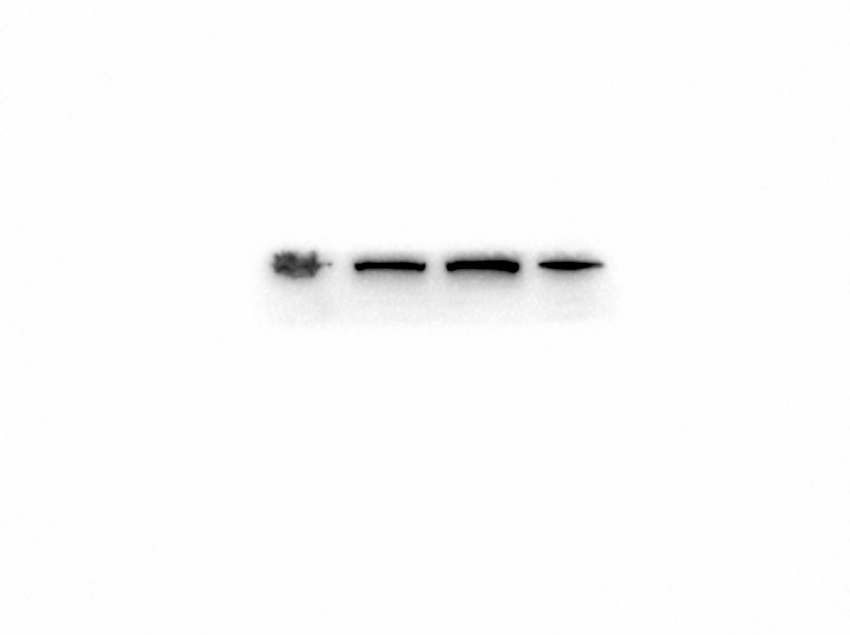

Supplement: Supplemental Information 7 [file peerj-09-10848-s007.zip › Full-length_uncropped_blots/Figure 4/Figure 4B NFIB2.tif]

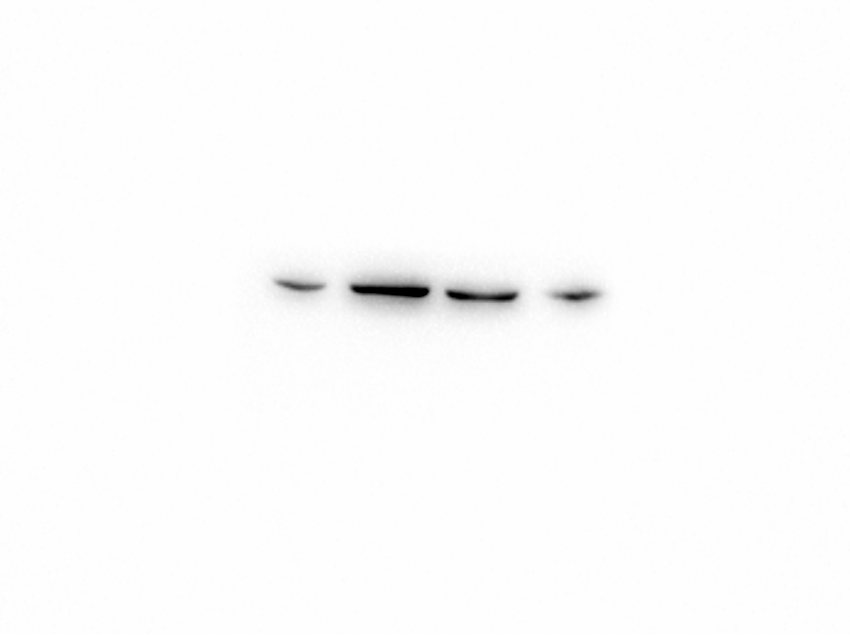

Supplement: Supplemental Information 7 [file peerj-09-10848-s007.zip › Full-length_uncropped_blots/Figure 4/Figure 4B PINK1.tif]

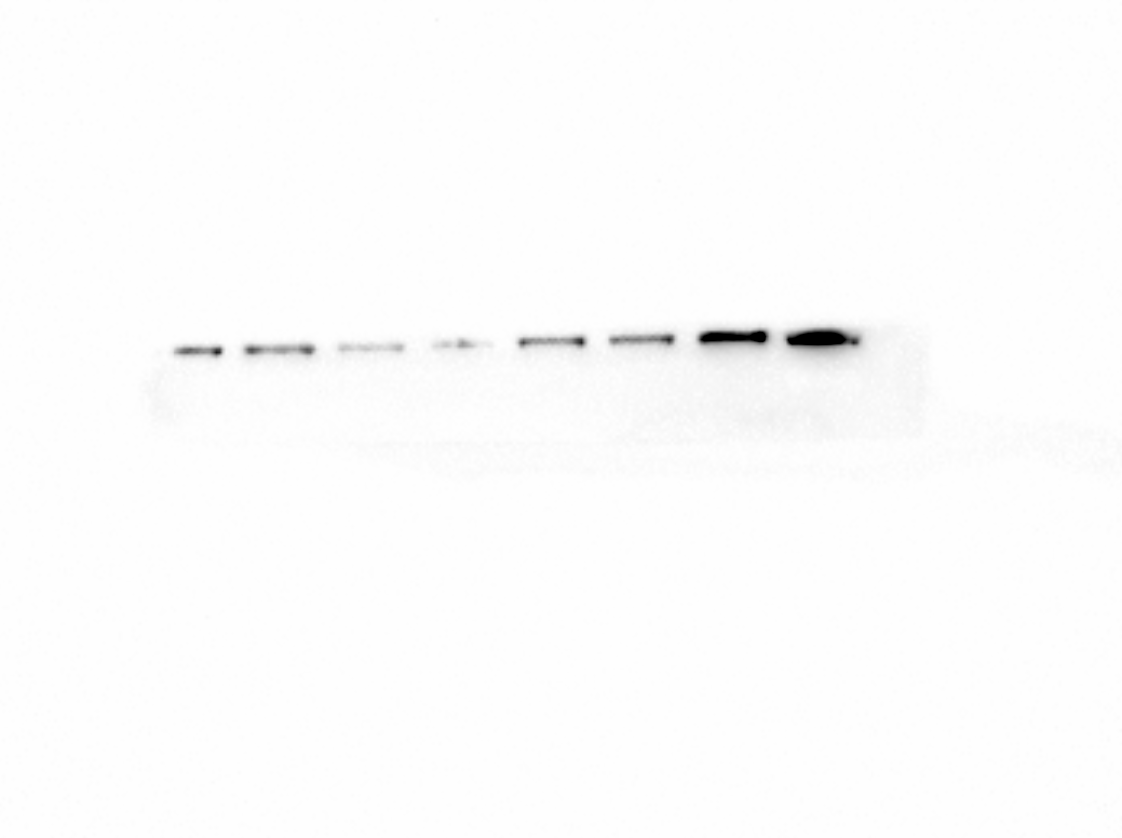

Supplement: Supplemental Information 7 [file peerj-09-10848-s007.zip › Full-length_uncropped_blots/Figure 4/Figure 4B GAPDH2.tif]

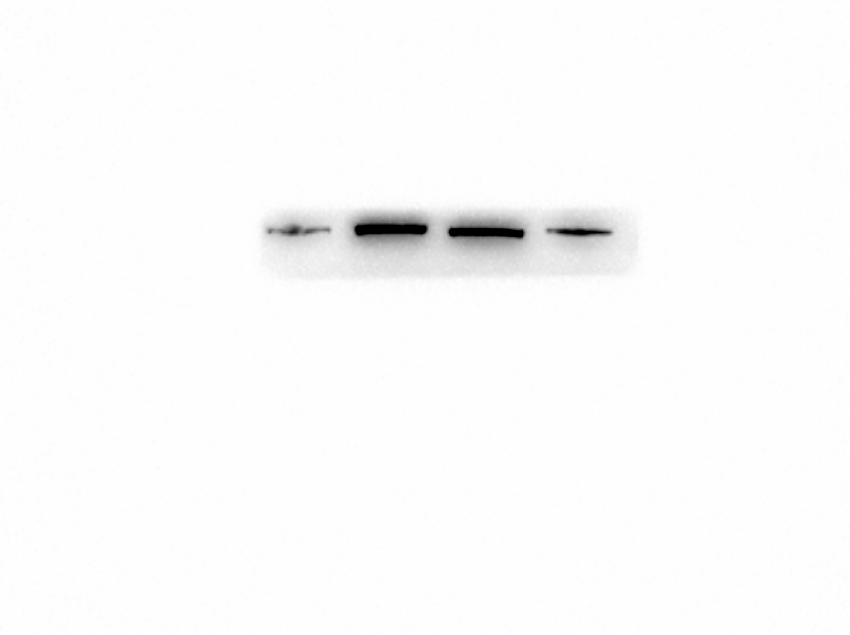

Supplement: Supplemental Information 7 [file peerj-09-10848-s007.zip › Full-length_uncropped_blots/Figure 4/Figure 4B NFIB.tif]

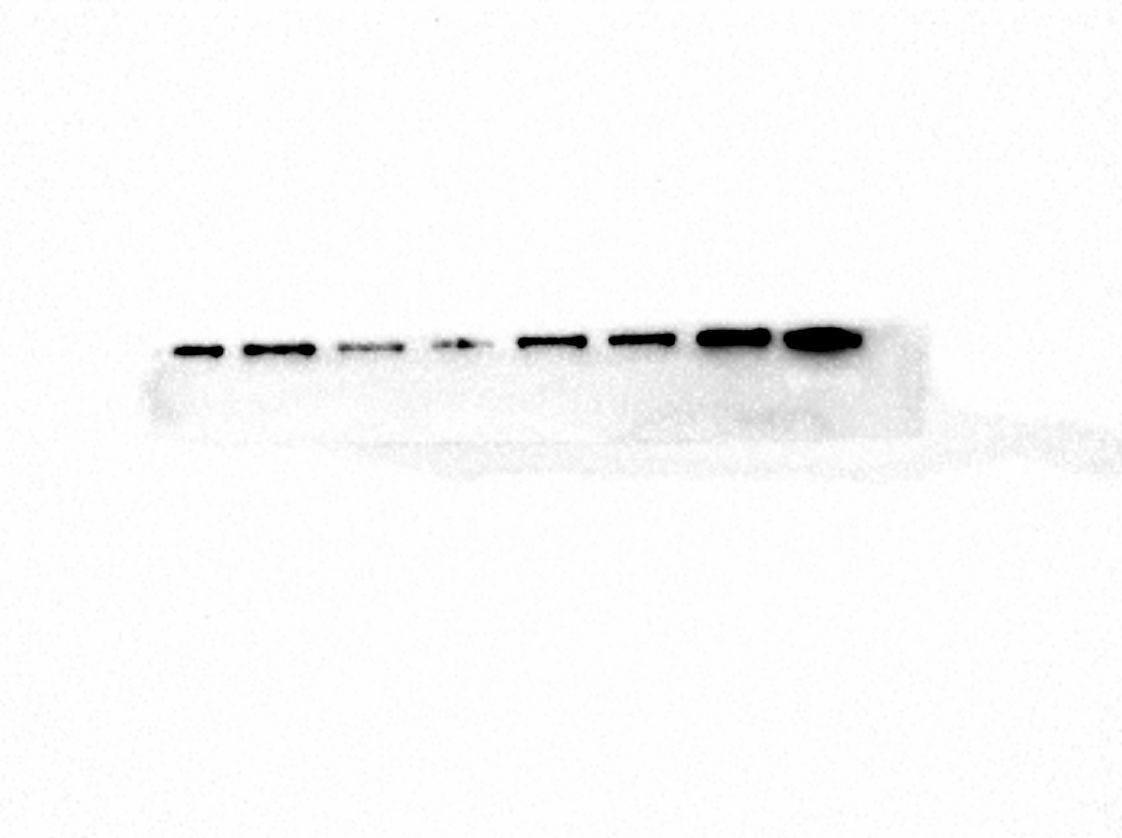

Supplement: Supplemental Information 7 [file peerj-09-10848-s007.zip › Full-length_uncropped_blots/Figure 4/Figure 4B GAPDH.tif]

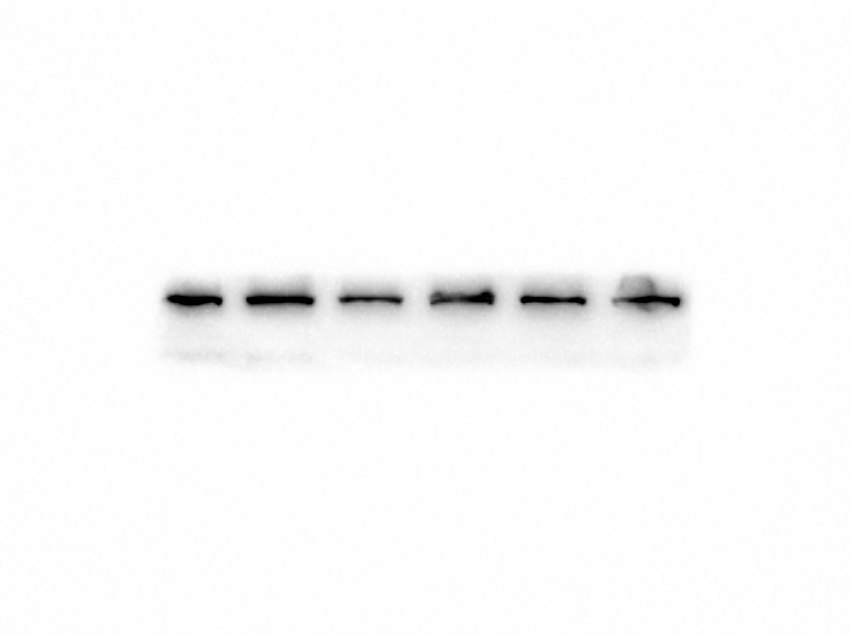

Supplement: Supplemental Information 7 [file peerj-09-10848-s007.zip › Full-length_uncropped_blots/Figure 4/Figure 4B PINK1 .tif]

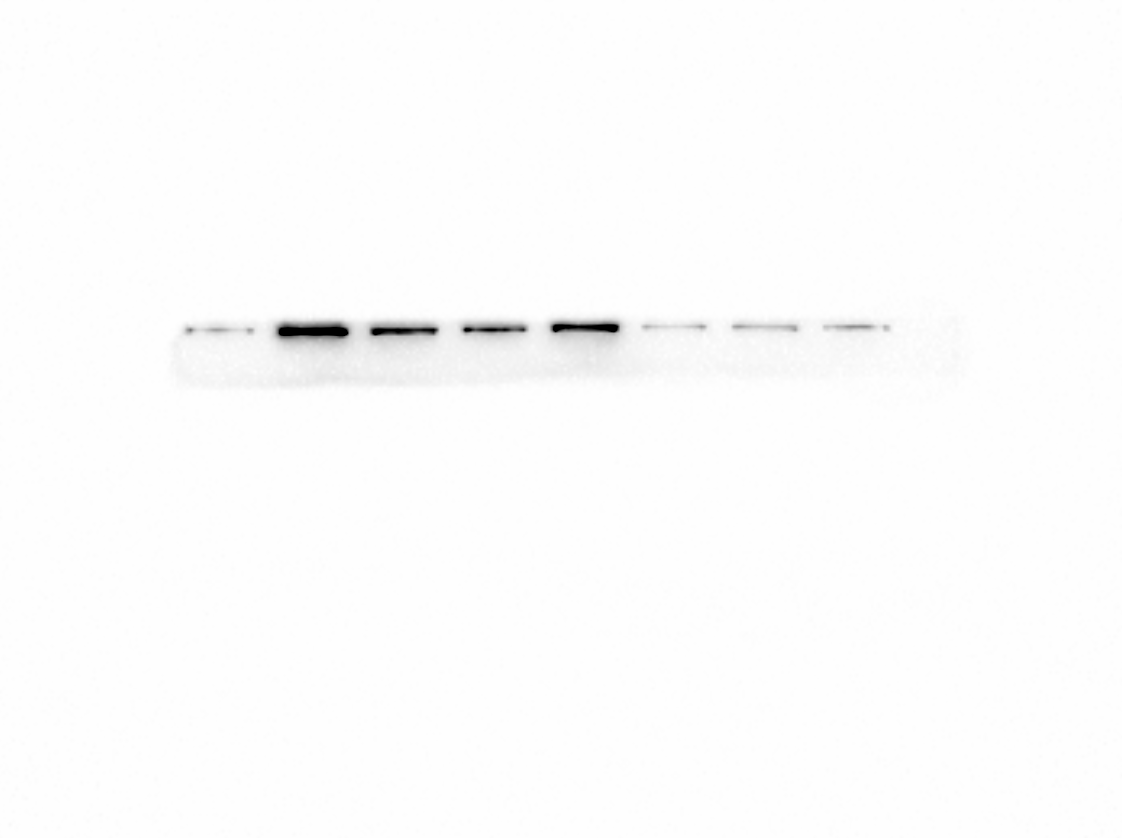

Supplement: Supplemental Information 7 [file peerj-09-10848-s007.zip › Full-length_uncropped_blots/Figure 3/Figure 3D NFIB.tif]

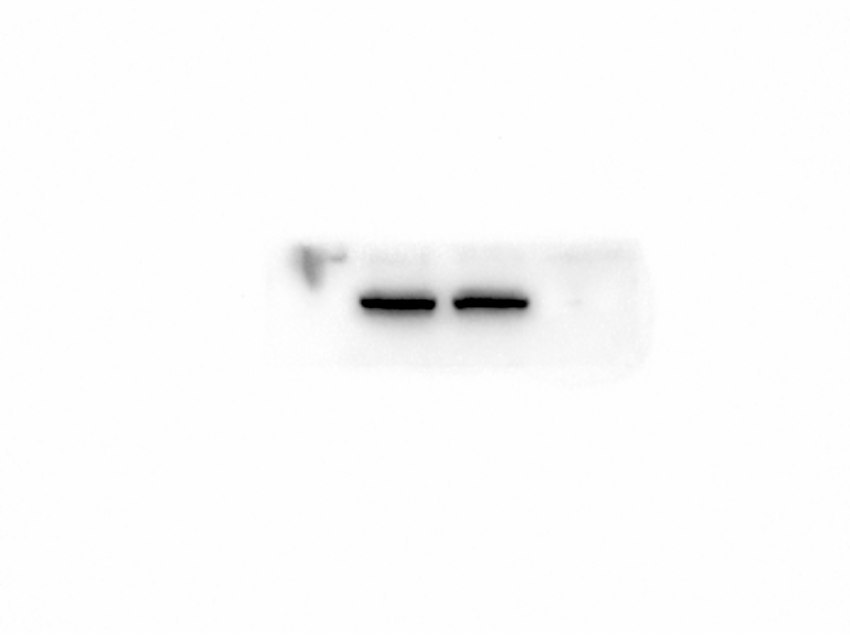

Supplement: Supplemental Information 7 [file peerj-09-10848-s007.zip › Full-length_uncropped_blots/Figure 3/Figure 3D GAPDH.tif]

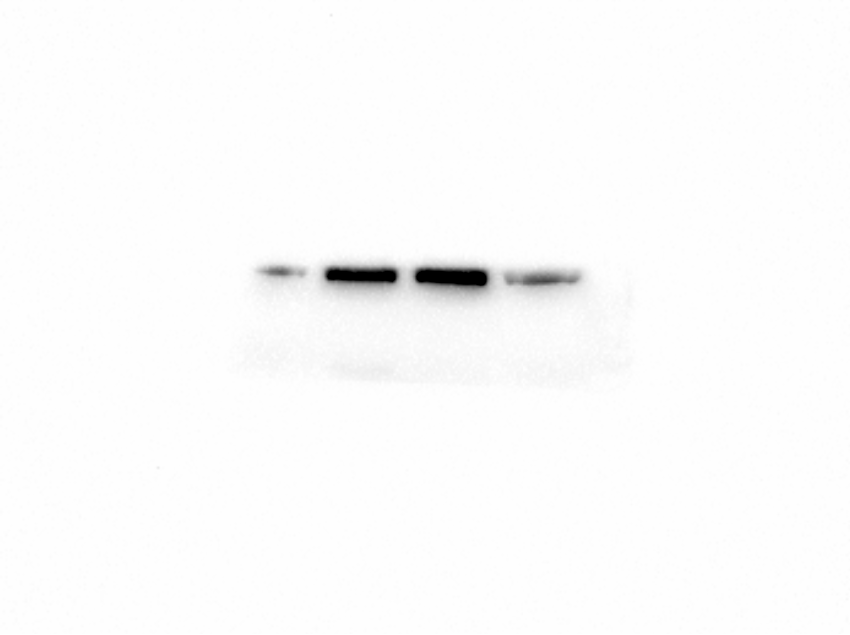

Supplement: Supplemental Information 7 [file peerj-09-10848-s007.zip › Full-length_uncropped_blots/Figure 3/Figure 3D GAPDH2.tif]

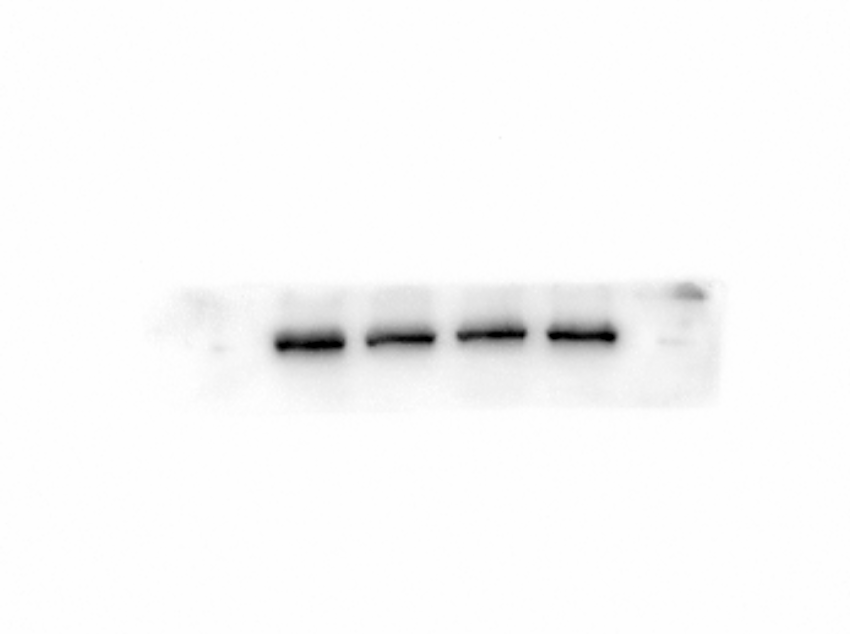

Supplement: Supplemental Information 7 [file peerj-09-10848-s007.zip › Full-length_uncropped_blots/Figure 3/Figure 3D PINK1.tif]

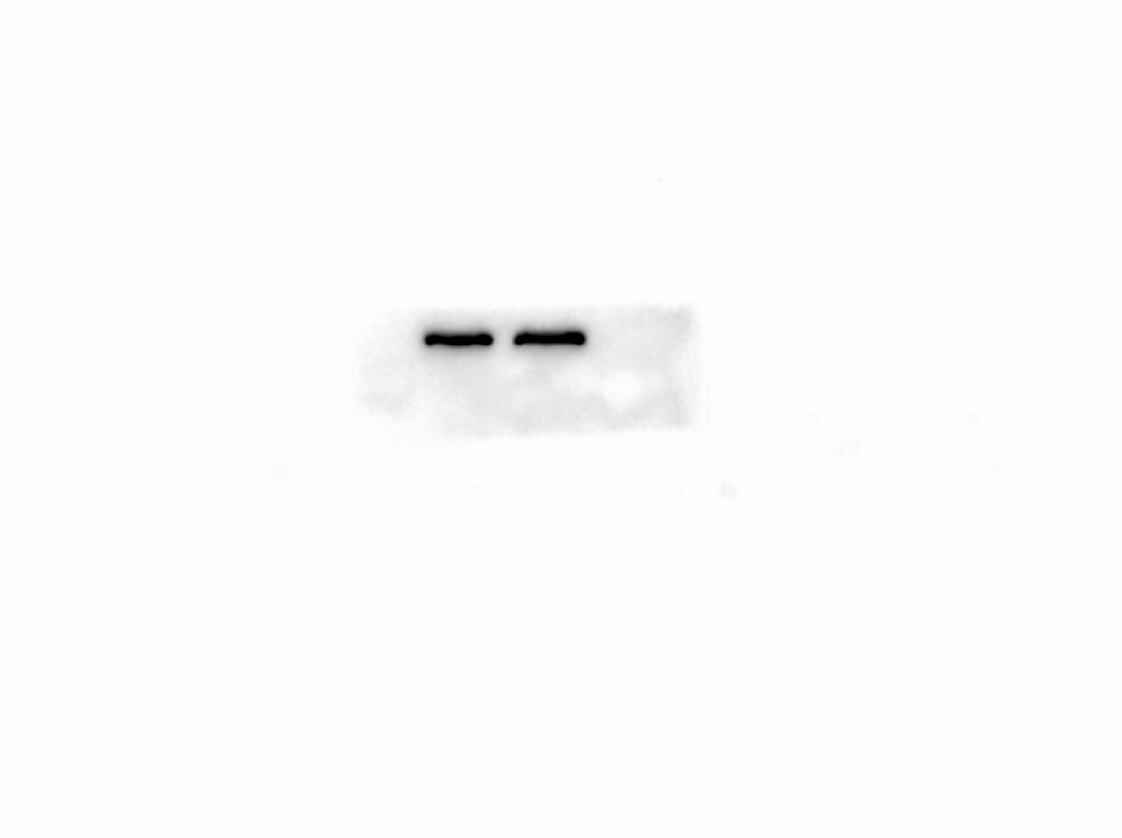

Supplement: Supplemental Information 7 [file peerj-09-10848-s007.zip › Full-length_uncropped_blots/Figure 3/Figure 3F GAPDH.tif]

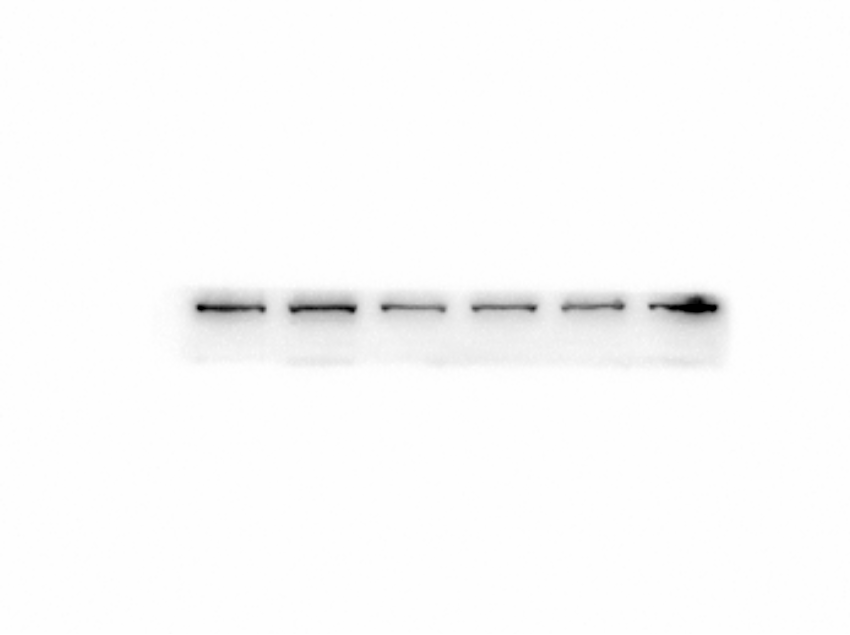

Supplement: Supplemental Information 7 [file peerj-09-10848-s007.zip › Full-length_uncropped_blots/Figure 3/Hepg2 CAS9-HDLBP.tif]

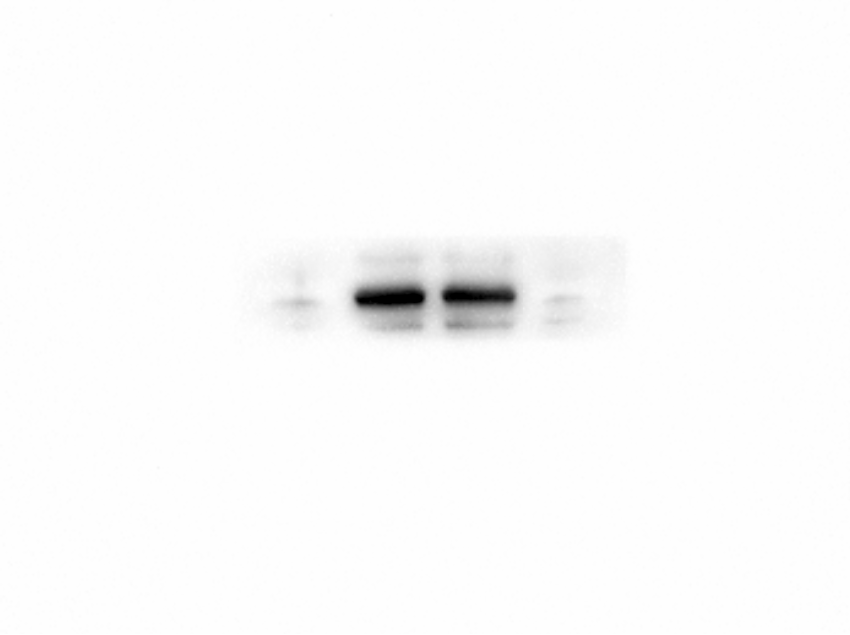

Supplement: Supplemental Information 7 [file peerj-09-10848-s007.zip › Full-length_uncropped_blots/Figure 3/Figure 3D GAPSH2.tif]

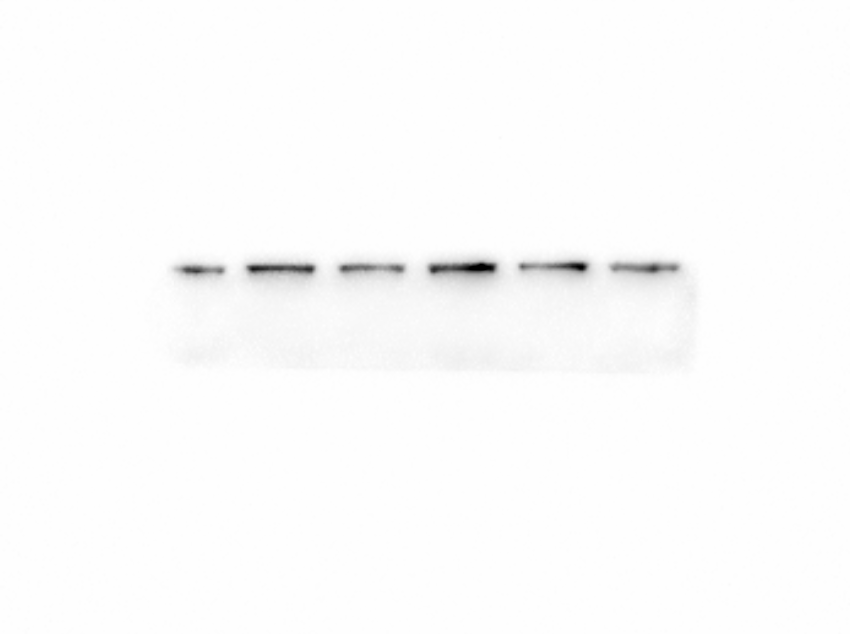

Supplement: Supplemental Information 7 [file peerj-09-10848-s007.zip › Full-length_uncropped_blots/Figure 3/Hepg2 CAS9-Braf.tif]
